# Supplementary material for: Simplest mechanism builder algorithm (simba): an automated microkinetic model discovery tool
Source: Chem Sci. 2025 Aug 11;17(15):7533–50. doi: 10.1039/d5sc01473e (PMC12919723; doi:10.1039/d5sc01473e)
Supplement: SC-017-D5SC01473E-s001 [file SC-017-D5SC01473E-s001.pdf]

Supplementary Information (SI) for Chemical Science.
This journal is © The Royal Society of Chemistry 2026

Simplest Mechanism Builder Algorithm (SiMBA): An Automated Microkinetic Model Discovery Tool – S.I.

Miguel Ángel de Carvalho Servia
Department of Chemical Engineering
Imperial College London
South Kensington, London, SW7 2AZ, UK
m.de-carvalho-servia21@imperial.ac.uk

King Kuok (Mimi) Hii
Department of Chemistry
Imperial College London
White City, London, W12 0BZ, UK
mimi.hii@imperial.ac.uk

Klaus Hellgardt
Department of Chemical Engineering
Imperial College London
South Kensington, London, SW7 2AZ, UK
k.hellgardt@imperial.ac.uk

Dongda Zhang *
Department of Chemical Engineering
The University of Manchester
Manchester, M13 9PL, UK
dongda.zhang@manchester.ac.uk

Ehecatl Antonio del Rio Chanona *
Department of Chemical Engineering
Imperial College London
South Kensington, London, SW7 2AZ, UK
a.del-rio-chanona@imperial.ac.uk

August 13, 2025

A In-Depth Discussion of First Iteration of SiMBA for the Dehydration of Fructose

We have included in Table 1 all ten candidate matrices generated by SiMBA in the first iteration for the fructose dehydration case study. A close inspection of these matrices reveals that many are merely permuted or relabeled versions of one another and therefore produce identical dynamic behavior. For example, candidates 1 and 2 differ only by the interchange of the arbitrary intermediate labels “D" and “E", yet their corresponding ODE systems are algebraically the same once one renames $C_D \leftrightarrow C_E$. Likewise, candidates 2 and 3 represent the identical sequence of elementary steps presented in different row order, and again yield the same set of rate equations. The same equivalences occur between pairs (1, 4), (5, 6), (7, 9) and (8, 10).

These redundancies highlight a symmetry in the mechanism-generation phase: although SiMBA enforces stoichiometric and elementary-step constraints, it does not yet collapse equivalent representations that differ only by permutation of intermediates or step ordering. In practice this means computational effort is spent evaluating multiple “distinct" candidates that in fact behave identically. Recognizing this, future versions of SiMBA could incorporate additional canonicalization rules – such as enforcing an ordering on intermediate labels or elementary-step sequences – to prune these symmetric duplicates a priori. Doing so would streamline the search, ensuring that each unique dynamic behavior is explored only once.

Table 1: Candidate reaction matrices, mechanisms and ODE systems from iteration 1 for the fructose dehydration case study.

Candidate	Matrix Representation	Reaction Mechanism	ODE System
1	$\begin{bmatrix} -1 & 0 & 0 & 1 & 1 \\ 0 & 1 & 1 & -1 & 0 \\ 0 & 2 & 0 & 0 & -1 \end{bmatrix}$	$A \rightarrow D + E$<br>$D \rightarrow B + C$<br>$E \rightarrow 2B$	$\dot{C}_A = -k_1 C_A$<br>$\dot{C}_B = k_2 C_D + k_3 C_E$<br>$\dot{C}_C = k_2 C_D$<br>$\dot{C}_D = k_1 C_A - k_2 C_D$<br>$\dot{C}_E = k_1 C_A - k_3 C_E$
2	$\begin{bmatrix} -1 & 0 & 0 & 1 & 1 \\ 0 & 1 & 1 & 0 & -1 \\ 0 & 2 & 0 & -1 & 0 \end{bmatrix}$	$A \rightarrow D + E$<br>$E \rightarrow B + C$<br>$D \rightarrow 2B$	$\dot{C}_A = -k_1 C_A$<br>$\dot{C}_B = k_2 C_E + k_3 C_D$<br>$\dot{C}_C = k_2 C_E$<br>$\dot{C}_D = -k_1 C_A - k_3 C_D$<br>$\dot{C}_E = k_1 C_A - k_2 C_E$
3	$\begin{bmatrix} -1 & 0 & 0 & 1 & 1 \\ 0 & 2 & 0 & -1 & 0 \\ 0 & 1 & 1 & 0 & -1 \end{bmatrix}$	$A \rightarrow D + E$<br>$D \rightarrow 2B$<br>$E \rightarrow B + C$	$\dot{C}_A = -k_1 C_A$<br>$\dot{C}_B = k_2 C_D + k_3 C_E$<br>$\dot{C}_C = k_3 C_E$<br>$\dot{C}_D = k_1 C_A - k_2 C_D$<br>$\dot{C}_E = k_1 C_A - k_3 C_E$
4	$\begin{bmatrix} -1 & 0 & 0 & 1 & 1 \\ 0 & 2 & 0 & 0 & -1 \\ 0 & 1 & 1 & -1 & 0 \end{bmatrix}$	$A \rightarrow D + E$<br>$D \rightarrow 2B$<br>$D \rightarrow B + C$	$\dot{C}_A = -k_1 C_A$<br>$\dot{C}_B = k_2 C_E + k_3 C_D$<br>$\dot{C}_C = k_3 C_E$<br>$\dot{C}_D = k_1 C_A - k_3 C_D$<br>$\dot{C}_E = k_1 C_A - k_2 C_E$
5	$\begin{bmatrix} -1 & 0 & 1 & 0 & 1 \\ 0 & 1 & 0 & 1 & -1 \\ 0 & 2 & 0 & -1 & 0 \end{bmatrix}$	$A \rightarrow C + E$<br>$E \rightarrow B + D$<br>$D \rightarrow 2B$	$\dot{C}_A = -k_1 C_A$<br>$\dot{C}_B = k_2 C_E + k_3 C_D$<br>$\dot{C}_C = k_1 C_A$<br>$\dot{C}_D = -k_2 C_E - k_3 C_D$<br>$\dot{C}_E = k_1 C_A - k_2 C_E$
6	$\begin{bmatrix} -1 & 0 & 1 & 1 & 0 \\ 0 & 1 & 0 & -1 & 1 \\ 0 & 2 & 0 & 0 & -1 \end{bmatrix}$	$A \rightarrow C + D$<br>$D \rightarrow B + E$<br>$E \rightarrow 2B$	$\dot{C}_A = -k_1 C_A$<br>$\dot{C}_B = k_1 C_A$<br>$\dot{C}_C = k_1 C_A$<br>$\dot{C}_D = k_2 C_D - k_3 C_D$<br>$\dot{C}_E = k_2 C_D - k_3 C_E$
7	$\begin{bmatrix} -1 & 1 & 0 & 0 & 1 \\ 0 & 0 & 1 & 1 & -1 \\ 0 & 2 & 0 & -1 & 0 \end{bmatrix}$	$A \rightarrow B + E$<br>$E \rightarrow B + D$<br>$D \rightarrow 2B$	$\dot{C}_A = -k_1 C_A + k_3 C_D$<br>$\dot{C}_B = k_1 C_A + k_3 C_D$<br>$\dot{C}_C = k_2 C_E$<br>$\dot{C}_D = k_2 C_E - k_3 C_D$<br>$\dot{C}_E = k_1 C_A - k_2 C_E$
8	$\begin{bmatrix} -1 & 1 & 0 & 0 & 1 \\ 0 & 1 & 0 & 1 & -1 \\ 0 & 1 & 1 & -1 & 0 \end{bmatrix}$	$A \rightarrow B + E$<br>$E \rightarrow B + D$<br>$D \rightarrow B + C$	$\dot{C}_A = -k_1 C_A$<br>$\dot{C}_B = k_1 C_A + k_2 C_E + k_3 C_D$<br>$\dot{C}_C = k_3 C_D$<br>$\dot{C}_D = k_2 C_E - k_3 C_D$<br>$\dot{C}_E = k_1 C_A - k_2 C_E$

(continued on next page)

Table 1 continued

Candidate	Matrix Representation	Reaction Mechanism	ODE System
9	$\begin{bmatrix} -1 & 1 & 0 & 1 & 0 \\ 0 & 0 & 1 & -1 & 1 \\ 0 & 2 & 0 & 0 & -1 \end{bmatrix}$	$A \to B + D$<br>$D \to C + E$<br>$E \to 2B$	$\dot{C}_A = -k_1 C_A$<br>$\dot{C}_B = k_1 C_A + k_3 C_E$<br>$\dot{C}_C = k_2 C_D$<br>$\dot{C}_D = k_1 C_A - k_2 C_D$<br>$\dot{C}_E = k_2 C_D - k_3 C_E$
10	$\begin{bmatrix} -1 & 1 & 0 & 1 & 0 \\ 0 & 1 & 0 & -1 & 1 \\ 0 & 1 & 1 & 0 & -1 \end{bmatrix}$	$A \to B + D$<br>$D \to B + E$<br>$E \to B + C$	$\dot{C}_A = -k_1 C_A$<br>$\dot{C}_B = k_1 C_A + k_2 C_D + k_3 C_E$<br>$\dot{C}_C = k_3 C_E$<br>$\dot{C}_D = k_1 C_A - k_2 C_D$<br>$\dot{C}_E = k_2 C_D - k_3 C_E$
